# Cortical Gamma Oscillations: Details of Their Genesis Preclude a Role in Cognition

**DOI:** 10.3389/fncom.2016.00078

**Published:** 2016-07-27

**Authors:** Bjorn H. Merker

**Affiliations:** Kristianstad, Sweden

**Keywords:** binding-by-synchrony, cognition, communication-through-coherence, cycle-by-cycle balancing, degrees of freedom, excitation-inhibition balance, gamma oscillations

Oscillatory activity in the gamma range (30–100 Hz, see further below) has repeatedly been proposed as a modality of cognitive operations in the cortex on account of its covariation with a number of cognitive variables. I recently reviewed this literature (Merker, 2013), concluding that the evidence is compatible with a far more modest view of the functional role of gamma, long known to result from the need to balance excitation with inhibition during cortical activation (Bartos et al., 2007). Gamma will accordingly occur when cortical tissue is functionally activated, a fact that may suffice to account for its covariation with cognitive variables. The case of gamma would accordingly be analogous, albeit on a finer time scale, to the way the BOLD signal covaries with cognitive variables without for that reason performing cognitive operations. On this “infra-structural” interpretation of gamma activity (see Merker, 2013, for details), its tight correlation with the BOLD signal is expected, and is roundly confirmed empirically (Chawla et al., 1999; Logothetis et al., 2001; Mukamel et al., 2005; Niessing et al., 2005; Lachaux et al., 2007; Nir et al., 2007; Zaehle et al., 2009; Jerbi et al., 2010; Ossandón et al., 2011; Scheeringa et al., 2011). Here I extend this perspective on gamma oscillations by showing that they are precluded from performing cognitive operations by the specifics of how their underlying physiology balances excitation with inhibition.

The principal cells of the cerebral cortex—its pyramidal cells—are exclusively excitatory, using glutamate as their transmitter. Some 4/5 of the thousands of synapses that stud their surface are excitatory, and most of these afferents derive from hundreds to thousands of other pyramidal cells located near and far in the cortical expanse (for details, see Peters, 1987a,b; Douglas and Martin, 1991; Beaulieu et al., 1992; Braitenberg and Schüz, 1998; Thomson and Lamy, 2007; Harris and Mrsic-Flogel, 2013; Callaway and Luo, 2015, pp. 8982–8983). Without further neuronal arrangements, this excitatory-to-excitatory self-connectivity of the cortex would be liable to runaway excitation in the form of epileptiform seizure activity at the network level (Jefferys, 1990; Wendling et al., 2000, 2002; Netoff et al., 2004; Buzsáki, 2006; Moore et al., 2010), and to the saturation of firing rates at the level of individual neurons (Shadlen and Newsome, 1994). These liabilities are remedied by a complement of roughly 20% cortical inhibitory neurons. Their processes are typically, though not always (see Caputi et al., 2013 and references therein), confined to their local vicinity. They interact with both principal cells and one another using GABA as their transmitter, and come in several kinds (Beaulieu, 1993; Binzegger et al., 2004; Ascoli et al., 2008; Burkhalter, 2008; Karnani et al., 2016).

The numbers, connectivity, and synaptic weights of these inhibitory auxiliaries are such as to ensure excitation-inhibition balance: rising excitation recruits additional inhibitory interneurons to effect a matching rise in pyramidal cell inhibition (see e.g., Douglas and Martin, 1991; Anderson et al., 2000; Wehr and Zador, 2003; Haider et al., 2006; Okun and Lampl, 2008). So crucial is this balance to normal cortical operation that it is underwritten by homeostatic mechanisms that restore it under persistent perturbation (Le Roux et al., 2006). The process is so specific that it adjusts inhibitory weights arriving from different interneurons onto pyramidal cells in accordance with the activity levels of individual pyramidal cells, leaving roughly equal excitation-inhibition ratios across them (Xue et al., 2014). Cortical dynamic range, sensitivity, linearization, and gain control are all aspects of or dependent upon this basic interaction between excitation and inhibition (van Vreeswijk and Sompolinsky, 1996; Chance et al., 2002; Pouille et al., 2009; see also Rubin et al., 2015; Barron et al., 2016).

These circumstances bear directly on gamma oscillations and their interpretation in that the balancing of excitation with inhibition by the interaction of principal cells with their inhibitory auxiliaries causes the local circuit to oscillate for fundamental reasons. When exposed to excitatory drive, synaptic delays in the circuitry and the low-pass filtering introduced by the biophysics of neuronal transduction (membrane time constants, after-hyperpolarization, etc.), combine with neuronal gain (amplification) and the mutual interaction of its inhibitory neurons to generate oscillatory activity in the gamma range (Kirschfeld, 1991, 1992; Buhl et al., 1996, 1998; Cobb et al., 1997; Whittington et al., 2000, 2010; Buzsáki, 2006; Bartos et al., 2007). This occurs even in isolated pieces of cerebral cortex maintained in a tissue bath (Llinas et al., 1991; Buhl et al., 1998).

These “inhibition-based rhythms” (Whittington et al., 2000) constitute gamma as classically defined (Adrian, 1942; Bressler and Freeman, 1980). They exhibit one or two well defined frequency peaks in the power spectrum between 30 and 100 Hz with most of their spectral power typically confined to around half an octave around a frequency peak (Ray and Maunsell, 2011). They must not be confused, in other words, with the very different phenomenon called “high gamma” by some investigators (100–200 Hz; see Crone et al., 2006). As shown by Ray and Maunsell (2011), the latter is a broad-band phenomenon composed of spectral (Fourier) components of neural spike transients, for which the designation “high frequency oscillations” used by some investigators appears appropriate (Tort et al., 2013).

At the membrane level of principal cells, gamma episodes appear as a tonic depolarization countered by waves of inhibitory postsynaptic potentials at gamma frequency, causing action potentials to concentrate to the waning phase of inhibition (Burchell et al., 1998; Whittington et al., 2000). Crucially, as shown by simultaneously monitoring the membrane potentials of pairs of pyramidal cells—clamped to the reversal potential for exitation and inhibition, respectively—the amplitude and spacing of waves of excitation-inhibition, registered as gamma cycles in the local field potential, track, and match fluctuations in excitatory membrane drive on a cycle by cycle basis (Atallah and Scanziani, 2009). Gamma oscillations do not, in other words, resemble sinusoids of steady frequency for a given oscillatory burst. Instead they have a fluctuating waveform whose amplitude and frequency changes from one cycle to the next, driven by the changes in excitatory drive the underlying cellular events are busy balancing.

In this immediate inhibitory echoing of excitatory magnitude, a gamma cycle with a larger amplitude typically is followed by a longer interval to the next cycle. Occurring from one cycle to the next, this amounts to a change in both instantaneous frequency and phase, establishing a linkage between changes in amplitude, frequency and phase across individual gamma cycles. The *in vivo* recordings reported by Atallah and Scanziani have a mean gamma frequency of 35 Hz, around which cycle lengths vary from 12 to over 40 ms. This cycle length difference (>28 ms) spans the full 29 ms phase range (2 Pi radians) of the oscillatory mean frequency of 35 Hz. Taken together, these circumstances harbor profound implications for our conception of the functional significance of gamma oscillations, implications which it has fallen upon me to spell out here for the first time, as follows:

The three principal dimensions or parameters characterizing an oscillatory phenomenon are frequency, amplitude, and phase. As just noted, the cellular events ensuring excitation-inbibition balance in the cortex are reflected in cycle by cycle changes in all three of these parameters of the local field potential gamma rythm. With gamma amplitude, instantaneous frequency, and phase all tied up in the basics of balancing excitation with inhibition from moment to moment, *no substantive degrees of freedom remain by which additional informational dimensions - such as cognitive ones - might be reflected in or carried by cortical gamma*.

But, objects the critic accustomed to thinking of gamma as a cognitive operator of some kind, what of the innumerable specific correlations reported over the years between gamma and cognitive variables? As already mentioned, and covered in greater detail in Section 6 of my 2013 review, there is no contradiction between such findings and the present interpretation of gamma. On the contrary, every one of those correlations is expected because cortical gamma signals an activated state of cortical tissue: cognitive activity of the most diverse kinds involve cortical activation. By exact analogy to the BOLD signal, the *specificity* of gamma covariation with cognitive variables does not belong to gamma, but to *where in cortical space* that activation is recorded. It is electrode location, in keeping with basic principles of functional specialization across the cortical sheet (e.g., Woolsey, 1947; Passingham et al., 2002), that accounts for cognitive and task specificity, while the occurrence of gamma at that location merely signals its state of activation. The same is of course true of high frequency oscillations. As a robust signal of local activation state with better temporal resolution than the BOLD signal, gamma, and high frequency oscillations have obvious utility as a means of mapping functional activity in the cortex.

A critic may nevertheless go on to note that the entire argument so far has pertained to local events, yet a variety of long-range effects involving synchrony or coherence of gamma oscillations are documented in the literature (e.g., Varela et al., 2001; Fries, 2005; Womelsdorf et al., 2007; Vicente et al., 2008; Gómez-Gardeñes et al., 2010; Gregoriou et al., 2012; see also Caputi et al., 2013; Buzsáki and Schomburg, 2015). From the present perspective these effects, including “communication-through-coherence” described by Fries (2005), are unproblematic, however. They arise as direct consequences of cortical connectivity. Axonal projections within areas, between areas, and between the hemispheres convey oscillatory rhythms to their targets to the extent that their axon potentials are grouped by oscillatory activity at their source (see Sections 4, 5 of Merker, 2013). Under circumstances of reciprocal connectivity and shared input from a third source, distant loci may even exhibit synchrony with zero phase lag (Chawla et al., 2001; Rajagovindan and Ding, 2008; Vicente et al., 2008).

Commonly, however, delays and phase shifts are involved in distance effects because the details of connective relations, such as differential laminar disposition, constrain coherence effects (see, e.g., Bastos et al., 2015 and references therein). The enhanced efficacy of synchronized afferents at a target structure is also no mystery. It conforms to elementary contingencies of summation at the axon initial segment: afferents arriving at a given dendritic tree close together in time have a higher probability of summing to an action potential at its axon initial segment than do those that are not clustered in time (Rall, 1962; Nowak and Bullier, 1997; see also Section 4, Merker, 2013).

The circumstances pertaining to gamma oscillations and their genesis reviewed here in all brevity are compatible with the empirically demonstrated behavior of these oscillations, but sharply contradict the pervasive tendency to attribute cognitive significance to their occurrence. Not only is there no more reason to do so in their case than in the case of the cortical BOLD signal (also ubiquitously covarying with cognitive variables), but the engagement of all three dimensions of gamma variability - amplitude, instantaneous frequency, and phase - in its cycle by cycle reflection of excitation-inhibition balancing, would seem to preclude additional functional roles, whether of a cognitive or any other kind.

The pervasive tendency to cast cortical gamma in cognitive terms originated with the so called ‘binding by synchrony’ conjecture of Singer et al. (Gray et al., 1989; Singer and Gray, 1995). As was shown in detail in Section 5 of Merker (2013), the binding in that case is provided by well documented anatomical connectivity. The same is true for the more general case of how the brain manages the collation and conjunction (“binding”) of attributes defined on spatially separate cortical maps, as explicated most fully by Kawato in (1997, p. 242 and Figure 15.3). It would seem to be time, then, to retrace that first false step of 1989, to give up the counterfactual casting of gamma as a cognitive operator, and return it to its rightful place amidst the crucial infrastructural operations that balance excitation with inhibition when cortex engages functionally for any of its many reasons to do so.

## Author contributions

As sole author of an opinion I both conceived and wrote the contents of the submission

### Conflict of interest statement

The author declares that the research was conducted in the absence of any commercial or financial relationships that could be construed as a potential conflict of interest.
